# Pneumocephalus and headache following craniotomy during the immediate postoperative period

**DOI:** 10.1186/s12893-022-01701-0

**Published:** 2022-06-29

**Authors:** Tae Kwan Kim, Jun Rho Yoon, Yee Suk Kim, Yuna Choi, Seheui Han, Jaeuk Jung, Ik Seong Park

**Affiliations:** 1 Department of Anesthesiology and Pain Medicine, Bucheon St. Mary’s Hospital, College of Medicine, The Catholic University of Korea, Seoul, Republic of Korea; 2 Department of Neurosurgery, Bucheon St. Mary’s Hospital, College of Medicine, The Catholic University of Korea, Seoul, Republic of Korea; 3 Department of Anesthesiology and Pain Medicine, 3Eunpyeong St. Mary Hospital, College of Medicine, The Catholic University of Korea, Seoul, Republic of Korea

**Keywords:** Pneumocephalus, Craniotomy, Headache

## Abstract

**Background:**

Pneumocephalus may be responsible for post-craniotomy headache but is easily overlooked in the clinical situation. In the present study, the relationship between the amount of intracranial air and post-craniotomy headache was investigated.

**Methods:**

A retrospective observational study was performed on 79 patients who underwent minimal invasive craniotomy for unruptured cerebral aneurysms. Those who had undergone previous neurosurgery, neurological deficit before and after surgery were excluded The amount of air in the cranial cavity was measured using brain computed tomography (CT) taken within 6 h after surgery. To measure the degree of pain due to intracranial air, daily and total analgesic administration amount were used as a pain index. Correlation between intracranial air volume and total consumption of analgesic during hospitalization was tested using Spearman rank correlation coefficients. Receiver operating characteristics (ROC) analysis was used to determine the amount of air associated with increased analgesic consumption over 72 h postoperatively.

**Results:**

The mean amount of intracranial air was 15.6 ± 9.1 mL. Total administration of parenteral and oral analgesics frequency were 6.5 ± 4.5, 13.2 ± 7.9 respectively. A statically significant correlation was observed between daily and total parenteral analgesic consumption after surgery and the amount of intracranial air at followed-up brain CT postoperatively within 24 h (r = 0.69, p < 0.001), within 48 h (r = 0.68, p < 0.001), and total duration after surgery (r = 0.84, p < 0.001). The optimal cut-off value of 12.14 mL of intracranial air predicts the use of parenteral analgesics over 72 h after surgery.

**Conclusions:**

Pneumocephalus may be a causative factor for post-craniotomy pain and headache with surgical injuries.

## Background

Pain, especially headache, is a common occurrence following intracranial surgery and a clinically significant problem. Pain after craniotomy is typically throbbing and pounding, similar to a tension headache, and sometimes steady and continuous in the forehead [4, 6–8]. Post-craniotomy headache affects the quality of postoperative course. However, post-craniotomy headache has been neglected, underestimated, and poorly managed due to the belief of pain insensitivity in the brain parenchyma [15]. The exact pathophysiology of headache following craniotomy remains unknown, however, several causative factors including surgical trauma to scalp, muscles, and meninges, aseptic meningitis, nerve injury, and formation of neuroma in the surgical scar, have been suggested to contribute to post-craniotomy headache [15, 18].

Most pneumocephali after craniotomy are minimal and spontaneously resolve as air gets absorbed [20, 27]. However, a sufficient amount of air in the cranial cavity can occasionally be a potential cause of headache following craniotomy and is easily overlooked in many clinical situations [1]. Furthermore, the causal relationship between the amount of air and post-craniotomy headache has been investigated in only a few studies and a limited number of publications is available, mostly consisting of case reports and minimal clinical analysis [9]. In the present study, the association between the amount of intracranial air and postoperative headache after craniotomy was investigated.

## Methods

After approval from the Institutional Review Board of our hospital’s ethics committee (Institutional review board Catholic University of Korea Bucheon St. Mary’s Hospital, reference number = HC20RIS10055, and date of approval = 20200721), a retrospective observational study was conducted in patients who underwent minimally invasive craniotomy for unruptured cerebral aneurysm during a 3-year periods between June 1, 2016 and July 1, 2019. Informed consent was waived because of the retrospective nature of the study and the analysis used anonymous clinical data. Subjects who previously received a craniotomy or had neurological deficit before or after surgery were excluded. All craniotomies were performed under general anesthesia with standard monitoring including non-invasive and invasive blood pressure monitoring, peripheral oxygen saturation (SpO_2_), end tidal carbon dioxide (EtCO_2_) tension, and urine output and temperature monitoring. Anesthesia was induced by intravenous propofol or thiopental sodium, tracheal intubation facilitated with rocuronium bromide (Esmeron®), and maintained with oxygen-air-sevoflurane with continuous remifentanil infusion ranging from 0.05 to 0.2 mcg/kg/min. During surgery, the EtCO_2_ was maintained at 30–35 mmHg, invasive arterial blood pressure within 20% of preoperative values, and pulse oximetry and bispectral index (BIS) values were continuously monitored. All patients underwent minimally invasive craniotomy through trans-eyebrow or pterional route with the head rotated 15–30° to the opposite side depending on the location of aneurysm. Near the end of the operation, neurosurgeons filled the cranial dead space with saline to remove air.

After the operation, all patients were transferred to the neuro-intensive care unit (NS-ICU). The intensity of postoperative pain, including headache, was measured using a verbal numeric rating scale (NRS) every 4 h by trained NS-ICU nurses and level of consciousness based on the Glasgow coma scales recorded. The NRS scores range from 0 to 10, with 0 representing no pain and 10 representing the worst possible pain (mild pain, score 1–3; moderate pain, score 4–6; severe pain, score 7–10). For pain relief, neurosurgeons prescribed parenteral analgesics including non-steroidal anti-inflammatory drugs (ketorolac), tramadol, or opioids (pethidine, fentanyl) on an as-needed basis and to all patients with NRS score ≥ 4. On the day of surgery and the first postoperative day, ketorolac 30 mg was regularly administered twice per day if the NRS score was ≥ 4 or patients requested analgesics for pain relief after trained nurses determined the pain level on the NRS was ≥ 4. Then, on the second postoperative day, when patients were able to take oral medications, two tablets of oral analgesic (ULTRACET®-Tramadol Hydrochloride 75 mg/Acetaminophen 650 mg) were given regularly three times a day. Despite pain control with the above listed drugs, when patients complained regarding persistent pain and the NRS score was ≥ 4, oral analgesics such as acetaminophen, or tridol were given preferentially. Parenteral analgesics ketorolac 30 mg, pethidine 25 mg, fentanyl 25 mcg, or tramadol 50 mg were administered in that order when pain was not able to be controlled with oral analgesics. The number of analgesics administered until the 3rd day after surgery was counted daily and estimated as an index of pain (Table 3).

All patients underwent routine brain computed tomography (CT) 6 h after surgery and the images archived in the hospital picture archiving communication system (PACS). The amount of intracranial air was measured from the CT scan image in PACS. The cross-sectional area of air density was manually traced as a region of interest and the area was automatically calculated in the PACS and presented as cm^2^. The total amount of intracranial air was estimated based on summation of cross-sectional area in each CT slice.

Data were collected from the electronic medical records of patients. Patient-related factors included age, sex, weight, medical history (smoking, hypertension, diabetes mellitus, dyslipidemia), anesthesia-related factors such as the American Society of Anesthesiologist (ASA) classification, hospital stay, and surgical procedure data including surgical approaches (supraorbital or pterional) and duration of surgery.

### Statistical analysis

The statistical analysis was performed using SAS version 9.4 (SAS Institute, Cary, NC, USA). Continuous variables were expressed as mean ± standard deviation (SD) and categorical variables were counted in numbers (percentages values in parentheses, %). Correlation between the daily and total analgesic use, total duration of analgesic use from the day of surgery to discharge day, and the amount of intracranial air were analyzed using Spearman rank correlation coefficients (r). Bifrontal pneumocephalus was compared with unifrontal pneumocephalus using Wilcoxon rank sum test. A receiver operating characteristics (ROC) analysis was performed to assess whether greater analgesic use over a 48-h period was associated with increased intracranial air volume (pneumocephalus-related headache). The area under the ROC curve was calculated as well as sensitivity and specificity, and optimal cut-off values were determined using Youden index on ROC analysis. P-value < 0.05 was considered statically significant.

## Results

**Fig. 1:**
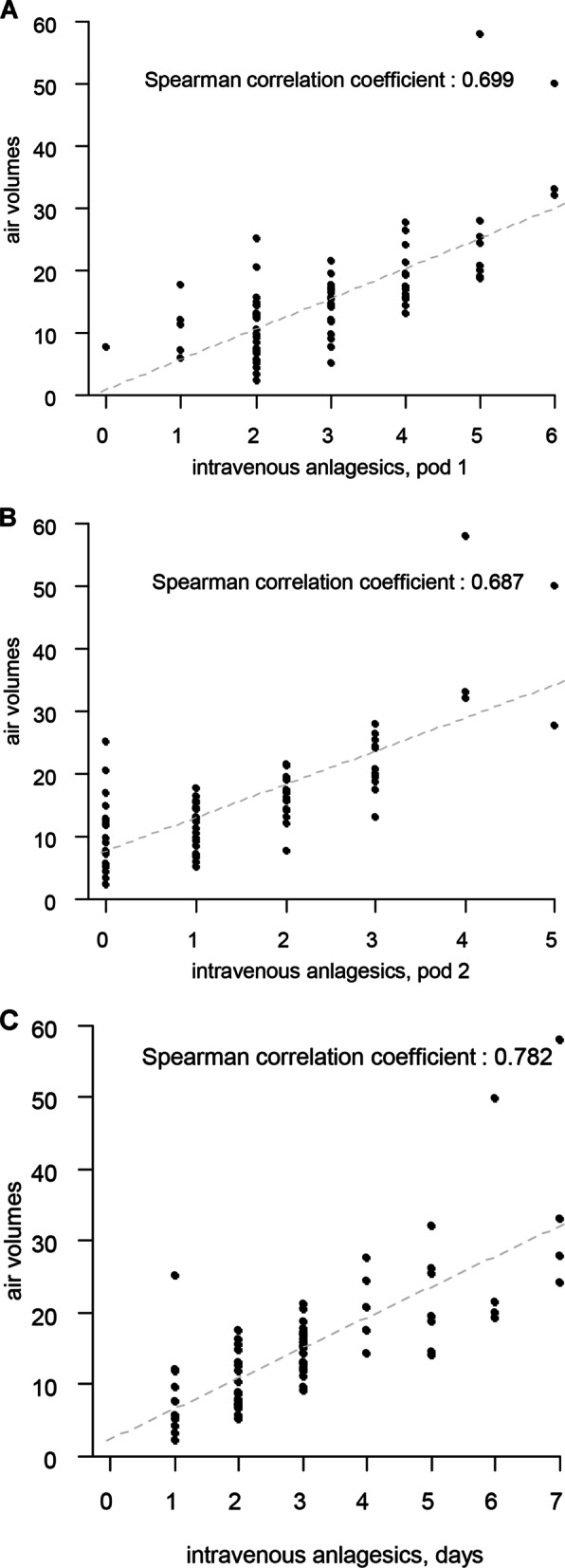
Scatter plot showing Spearman positive correlation of intracranial air volume and intravenous analgesic consumption **a** within 24 h after surgery (*r* = 0.699, p < 0.001), **b** within 48 h after surgery (*r* = 0.687, p < 0.001) and, **c** within 72 h after surgery (*r* = 0.726, p < 0.001)

**Fig. 2:**
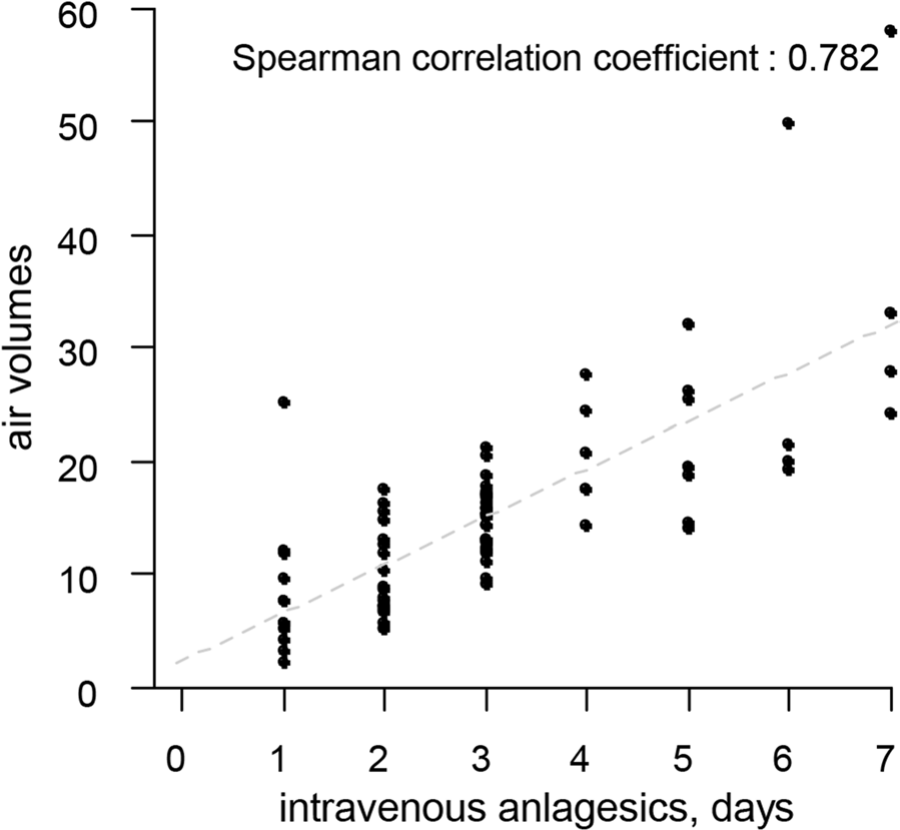
Scatter plot showing Spearman positive correlation of the total duration of parenteral analgesic consumption increased with increased amount of intracranial air (r = 0.782, p < 0.001)

A total of 103 patients were enrolled in this study, however, 24 patients were excluded during the study period due to the following reasons: postoperative irritability or delirium (9), inappropriate response to pain estimation or pain expression (7), wound abscess (1), postoperative hyponatremia with syndrome of inappropriate diuretic hormon, incomplete brain CT images (2), or incomplete medical records (4). A total of 79 consecutive patients were eligible for final analysis. Demographic characteristics are presented in Table 1. Changes in surgery-related complications which was able to cause headache were subdural fluid collection, scalp swelling and extradural hemorrhage. All of these complication was minimal and presented in Table 2. Table 3 shows the amount of air checked on brain CT 6 h after surgery, total amounts of analgesic consumption within 24 h, 48 h, 72 h. The total volume of intracranial air was 15.6 ± 9.1 mL (range 2.2–57.9 mL), the total parenteral analgesic consumption was 6.5 ± 4.5 (range 2.0–22), the average number of days of analgesic use was 3.1 ± 1.6 days, and the total oral analgesic consumption was 13.2 ± 7.9 (range 0–41). The administration frequency and duration of parenteral analgesic use increased in proportion to the amount of intracranial air. Figure 1 shows statistically significant correlation between parenteral analgesic use and amount of intracranial air from the day of surgery to day of discharge: within 24 h, within 48 h, and within 72 h after surgery (r = 0.699, 0.687, and 0.726, respectively; p < 0.001). In addition, the total duration of parenteral analgesic consumption increased with increased amount of intracranial air followed-up brain CT at 6 h after surgery (r = 0.782, p < 0.001) (Fig. 2). A statically significant difference was observed in the amount of air between unifrontal and bifrontal pneumocephalus (p < 0.001). The amount of air in unifrontal pneumocephalus was 11.3 ± 5.0 mL and in bifrontal pneumocephalus was 19.8 ± 10.1 mL. The ROC curve for parenteral analgesic use over 48 h showed the area under the curve (AUC) was 0.86 (95% CI 0.76–0.95) and the optimal cut-off value was 12.14 mL of intracranial air (sensitivity, 0.85%; specificity, 0.76%).

**Table 1:** Patient characteristics

	Total number of patients (%)
Sex	
Male	37 (46.8)
Female	42 (53.2)
Age (years)	53.5 ± 9.2
Weight (kg)	69.8 ± 34.3
ASA	
1	25 (31.7)
2	53 (67.1)
3	1 (1.3)
Operation approach	
Trans-eyebrow	40 (50.6)
Mini-pterional	39 (49.4)
Underlying disease	
Diabetes mellitus	
No	31 (39.2)
Yes	48 (60.8)
Hypertension	
No	35 (44.3)
Yes	44 (55.7)
Lipidemia	
No	58 (73.4)
Yes	21 (26.6)
Smoking	
No	63 (79.8)
Yes	16 (20.3)
Duration of operation (min)	187.1 ± 44.3
Postoperative hospital stay (days)	7.0 ± 2.3

Values are numbers (percentages) for categorical variables. Mean ± standard deviation (SD)

**Table 2:** Changes in surgery-related complications based on CT

CT findings	Total number of patients (%)	p-value
Subdural fluid collection		
No	69 (87.3)	0.92
Yes	10 (12.7)	
Eye swelling		
No	74 (93.7)	0.57
Yes	5 (6.3)	
Scalp swelling		
No	72 (91.1)	0.82
Yes	7 (8.9)	
Extradural hemorrhage		
No	70 (88.6)	0.67
Yes	9 (11.4)	
Other changes		
No	76 (96.2)	0.97
Yes	3 (3.8)	

Values are numbers (percentages) for categorical variables. P-value for difference was determined using Wilcoxon rank sum test

**Table 3:** Intracranial air volume and parenteral/oral analgesic administration frequency after surgery

	Mean	Min	Median	Max
Intracranial air volume (mL)	15.6 ± 9.1	2.2	14.4	57.9
Daily IV analgesics use frequency				
POD 24 h	3 ± 1.3	0	3	6
POD 48 h	1.5 ± 1.3	0	1	5
POD 72 h	1.1 ± 1.1	0	1	5
Total IV analgesic use frequency	6.5 ± 4.5	2	5	22
Daily oral analgesic use frequency				
ULTRACET®	9.2	0	9	41
Acetaminophen	3.5	0	0	31
Tridol	0.5	0	0	12
Total oral analgesic use frequency	13.2 ± 7.9	0	13	41

POD: postoperative day; POD 24 h: within 24 h after surgery; POD 48 h: within 48 h after surgery; POD 72 h: within 72 h after surgery; IV: intravenous

## Discussion

Pneumocephalus is commonly observed after intracranial surgery [3, 17, 22]. The incidence of pneumocephalus after supratentorial craniotomy was reportedly 100%, even in the supine position [3, 17, 22]. In the present study, pneumocephalus after craniotomy was common and variable amount of intracranial air was observed in the retrospectively reviewed CT images after surgery. This finding is consistent with previous observation that pneumocephalus was present in all patients within the first 2 postoperative days after supratentorial craniotomy [17]. Reasoner et al. observed that all patients had pneumocephalus immediately after supratentorial craniotomy based on retrospective analysis of CT findings in 240 patients, even in the non-sitting position. The authors reported that 66% of the pneumocephalus was estimated to be moderate or larger and this decreased to 75.0%, 59.6%, and 26.3% by 1, 2, and 3 weeks after surgery, respectively [13]. The authors also reported the total amount of intracranial air may vary, is benign in nature, and requires approximately 2–3 weeks for complete reabsorption [17, 22]. Previously, Toung et al. [25] observed that patient position influenced the incidence of intracranial air collection and varied from 73% for park-bench to 57% for prone, and 100% in the sitting position in patients who had undergone posterior or upper cervical cord procedures in the sitting position. This difference is probably associated with the amount of cerebrospinal fluid (CSF) drainage due to the patient’s head position during surgery [25].

Several factors may contribute to the development of pneumocephalus during craniotomy such as nitrous oxide administration (anesthesia), conditions which reduce the brain bulk including use of osmotic diuretics, moderate hyperventilation with subsequent hypocapnea, and subsequent drain of CSF [3, 22]. The development of pneumocephalus after craniotomy can be explained by a reduction in intracranial pressure and the presence of a defect in the dura and skull [21]. During surgery, the continuous loss of CSF results in intracranial hypotension compared with the atmosphere, allowing entrapment of air into the intracranial cavity through the cranial-dura defect [21, 27]. Air enters as a bubble, replacing the CSF as the pressure in the two cavities equilibrate [3].

In the present study, the volume of air varied from 2 to 59.7 mL (mean 15.7 ± 9.1 mL) based on the computed volume of air using head CT scans, which can detect as little as 0.5 mL air [3, 17]. Most pneumocephali were more common in the subdural space over the frontal lobe or both lobes as previously reported in other studies [3, 16]. The total amount of intracranial air was estimated based on summation of the cross-sectional area in each CT slice. This method of CT volume determination has been shown to be within an acceptable range of accuracy on phantoms and body organs in vitro with mean percentage error from 3.59 to 4.95% [16].

Acute headache following craniotomy is a frequent complaint due to surgical procedures and meningeal irritations [6]. Previously, craniotomy was generally considered to be less painful than other procedures due to absence of nociceptors in brain parenchyma and innervation limited to meninges, pericranial muscles, and fascia [6]. However, in contrast with this belief, pain following craniotomy was reported in several observational studies to be more common for the first 2 days after a major elective intracranial surgery with moderate to severe intensity [2, 4, 5, 7, 10, 11, 15, 18, 24, 26]. According to De Benneditt et al., approximately 60% of patients experienced moderate to severe pain after craniotomy for 48 h with maximum intensity 12 h postoperatively [4]. More recently, in a large prospective study, 69% of patients undergoing craniotomy experienced moderate to severe pain (NRS ≥ 4) on the first postoperative day and 48% of patients on the second postoperative day [10, 15].

Acute headaches following craniotomy within the first day are predominantly superficial (86%), indicating a somatic pain rather than visceral pain [4, 18]. The headache is usually localized to the incision site and surrounding area with maximal intensity at the incision site and pain intensity decreasing with time. Although most acute pain and headaches frequently occur within the first 48 h after craniotomy [2], a persistent postsurgical headache is observed in many patients [24]. Therefore, before surgery, neurosurgeons explain this potential problem and should get informed permission from the patients The proposed possible causes are dura irritation by air, muscle retraction, surgical trauma, decreased CSF pressure, and aseptic meningitis [15]. Acute pain mainly results from consequences of surgical incision of scalp and soft tissues or pericranium. However, dura manipulation during surgery also activates pain pathways and contributes to postoperative pain [2]. Mechanical and chemical irritation of dura remaining after surgery can lead to painful postoperative sensation as well as surgical trauma, even after effective scalp block [12]. The intensity of postoperative pain is more associated with the amount of muscle damage than the surgical location relative to the tentorium (i.e., supra or infra surgical approaches) [23, 24]. In the current study, small craniotomy was performed to preserve unnecessary extracranial muscle incisions using trans-eyebrow or mini-pterional approaches.

Although pneumocephalus after craniotomy is clinically asymptomatic and spontaneously resolved as air gets absorbed, sometimes excessive volume can cause several symptoms such as headache, nausea, vomiting, irritability, lethargy, dizziness, or neurologic deficits [3]. Headache is the most frequent presentation of pneumocephalus [23]. In epidural block complications, pneumocephalus-related headaches can suddenly develop, are not relieved with posture, and very sensitive to movement, consistent with irritation of the meninges due to intracranial air motion. Post-craniotomy headache can be caused by intracranial air causing dura irritation [1]. An intracranial air bubble behaves like a space-occupying lesion causing meningeal irritation. These symptoms usually recover within 5–7 days with intracranial air absorbed. The relationship between volume of air and headache occurrence has been investigated in only a few studies. Although in previously reported cases, pneumocephalus can cause severe headache, the amount of volume necessary to induce a headache has been evaluated in only a few studies [1]. The amount of intracranial air necessary to cause headache in spinal anesthesia varies, however, usually more than 20 mL in a sitting position is necessary [14] although significantly smaller amounts can cause headaches. According to a previous case report [19], as little as 2 mL of air in subarachnoid spaces can provoke a severe frontal headache and Hogan reported a case in which 2–4 mL of air in subdural spaces caused a headache [13]. Baker reported that headaches caused by subdural pneumocephalus were significantly more severe than other air distributions [1]. In the previous studies regarding volume in the intracranial cavity, asymptomatic and symptomatic tension pneumocephali were compared. Conversely, Monajatii and Cotanch cautioned that presence of more than 65 mL of air could result in tension pneumocephalus and 20 mL in asymptomatic pneumocephalus [16]. Contrary to this finding, Ishwata et al. [14] found no substantial difference in air volume between tension pneumocephalus and symptomatic pneumocephalus in chronic subdural hematoma.

In the present study, daily analgesic consumption decreased with time but the total duration of analgesic consumption was longer with greater volume of intracranial air. Differentiating the pain due to intracranial air from surgical injury in the acute period is not possible. To quantitatively analyze pain, the amount of analgesics rather than NRS score was used as an indicator of pain. The NRS score decreased after taking analgesics, thus, it did not reflect the degree of headache. In the present study, a statistically significant correlation between daily and total analgesic consumption and amount of intracranial air, especially bifrontal pneumocephalus, was observed compared with unifrontal pneumocephalus (Table 3). In addition, based on these observations, the total duration of analgesic consumption was longer with greater intracranial air accumulation. Consequently, the optimal cut-off value on ROC analysis showed 12.14 mL of intracranial air is more associated with pneumocephalus-related headache than acute post-craniotomy pain.

The present study had several limitations. First, consensus or standard treatments for pain control after craniotomy do not currently exist. Because neurosurgical patients require frequent neurologic examinations, excessive postoperative pain management may lead to an unintended risk of over-sedation of patients which could mask new neurologic deficits. Therefore suboptimal pain control using both oral and parenteral analgesics on an on-demand basis, especially oral analgesics, preferentially if possible, were provided in our study. In addition, patients undergoing craniotomy were treated with on-demand analgesic medication when the patient complained of pain ≥ 4 on NRS and were not allowed the same medication within 4 h after intravenous administration of drugs. Second, the potency of analgesics was not unified. Therefore, the effect of drugs may vary. Third, the presence of headache prior to surgery is an important risk factor for postoperative headache occurrence, as previously reported in several studies [4, 7, 8, 15], however this was not investigated in the present study. Instead, the patients who had unruptured aneurysms were included and received minimally invasive surgery with saline irrigation at end of the surgery.

## Conclusions

Acute postoperative pain and headache following craniotomy are important clinical issues but have been underestimated and poorly managed. Pneumocephalus is a frequently observed imaging finding on routine postoperative examination and may be a causative factor for post-craniotomy pain and headache with surgical injuries. To minimize the accumulation of intracranial air, several efforts including less CSF loss during surgery, filling the cranial cavity with saline as much as possible at the end of the surgery to avoid entrapment of air may be warranted.

## Data Availability

Not applicable because the data include patients profile.

## Abbreviations

ASA: American Society of Anesthesiologist
BIS: Bispectral index
CSF: Cerebrospinal fluid
CT: Computed tomography
NS-ICU: Neuro-intensive care unit
NRS: Numeric rating scale
PACS: Picture archiving communication system
ROC: Receiver operating characteristics
